# Physical activity, obesity and sedentary behavior in cancer etiology: epidemiologic evidence and biologic mechanisms

**DOI:** 10.1002/1878-0261.12772

**Published:** 2020-08-18

**Authors:** Christine M. Friedenreich, Charlotte Ryder‐Burbidge, Jessica McNeil

**Affiliations:** ^1^ Department of Cancer Epidemiology and Prevention Research CancerControl Alberta Alberta Health Services Calgary AB Canada; ^2^ Department of Oncology Cumming School of Medicine University of Calgary Calgary AB Canada; ^3^ Department of Community Health Sciences Cumming School of Medicine University of Calgary Calgary AB Canada

**Keywords:** chronic inflammation, hormone, metabolism, obesity, physical activity, sedentary behavior

## Abstract

An estimated 30–40% of cancers can be prevented through changes in modifiable lifestyle and environmental risk factors known to be associated with cancer incidence. Despite this knowledge, there remains limited awareness that these associations exist. The purpose of this review article was to summarize the epidemiologic evidence concerning the contribution of physical activity, sedentary behavior, and obesity to cancer etiology and to provide an overview of the biologic mechanisms that may be operative between these factors and cancer incidence. Strong and consistent evidence exists that higher levels of physical activity reduce the risk of six different cancer sites (bladder, breast, colon, endometrial, esophageal adenocarcinoma, gastric cardia), whereas moderate evidence inversely associates physical activity with lung, ovarian, pancreatic and renal cancer, and limited evidence inversely correlates physical activity with prostate cancer. Sedentary behavior, independent of physical activity, has been shown to increase the risk of colon, endometrial, and lung cancers. Obesity is an established risk factor for 13 different cancer sites (endometrial, postmenopausal breast, colorectal, esophageal, renal/kidneys, meningioma, pancreatic, gastric cardia, liver, multiple myeloma, ovarian, gallbladder, and thyroid). The main biologic mechanisms whereby physical activity, sedentary behavior, and obesity are related to cancer incidence include an effect on endogenous sex steroids and metabolic hormones, insulin sensitivity, and chronic inflammation. Several emerging pathways related to oxidative stress, DNA methylation, telomere length, immune function, and gut microbiome are presented. Key recommendations for future research in both the epidemiology and biology of the associations between physical activity, sedentary behavior, obesity, and cancer risk are also provided.

## Graphical abstract

An estimated 4 in 10 cancers are preventable through modifiable risk factors. This review summarizes the epidemiologic evidence linking physical activity, sedentary behavior, and obesity with cancer risk. We explore the possible biologic mechanisms underpinning the associations between these lifestyle factors and cancer incidence, including effects on endogenous sex steroids and metabolic hormones, insulin sensitivity, and chronic inflammation.

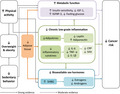

**Abbreviations**
BETA: Breast cancer and Exercise Trial in Alberta
BMI: body mass index
CRP: C‐reactive protein
IGF: insulin growth factor
IGFBP: insulin growth factor‐binding protein
IL‐1β: interleukin‐1 β
IL‐6: interleukin‐6
MET: metabolic equivalents of task
PAGA: Physical Activity Guidelines for Americans
RCT: randomized controlled trial
ROS: reactive oxygen species
RR: relative risk
SAA: serum amyloid A
SHBG: sex hormone‐binding globulin
TNF‐α: tumor necrosis factor‐α
UV: ultraviolet
WCRF/AICR: World Cancer Research Fund/American Institute for Cancer Research

## 1 Introduction

The epidemiologic evidence base regarding the etiologic role for physical activity, sedentary behavior, and obesity in cancer incidence has been evolving rapidly over the past three decades, and there is now convincing evidence for these associations. Research has also been conducted to examine the underlying biologic mechanisms that could explain how these risk factors are associated with increased cancer risk. Estimates of the population burden associated with modifiable risk factors and cancer incidence have demonstrated that 30–40% of cancers are potentially preventable [1, 2, 3, 4] and that some of the major risk factors associated with cancer include physical inactivity, sedentary behavior, and obesity. Furthermore, there is a considerable economic cost that could be avoided by decreasing the prevalence of these modifiable risk factors [5]. At present, the global prevalence of inactivity as defined by low levels of physical activity, sedentary behavior, and obesity is high [6, 7]. Given that these risk factors are modifiable, there is considerable potential to reduce the global burden of cancer through interventions targeting these factors. The aim of this paper was to provide an overview of the current epidemiologic evidence associating physical activity, sedentary behavior, and obesity with cancer incidence, and the hypothesized biologic mechanisms that are likely to connect these factors with cancer risk. The paper concludes with recommendations for future epidemiologic research on these topics to address some of the remaining knowledge gaps.

## 2 Physical activity and cancer incidence

Physical activity, defined as any bodily movement produced by skeletal muscles that requires energy expenditure, has been characterized and investigated in epidemiologic studies by the *domain* in which the activity is achieved (e.g., occupational, recreational, household, and transport activity), the *volume* of the activity (as measured by the frequency, duration, and intensity), and the *time periods* when the activity was done (ranging from current to lifetime activity). To date, over 500 observational epidemiologic studies have examined some aspect of the association between physical activity and cancer incidence. Most recently, this evidence has been evaluated and summarized for the Physical Activity Guidelines for Americans (PAGA) 2018 Report as well as by the World Cancer Research Fund/American Institute for Cancer Research (WCRF/AICR) as part of their recommendations on physical activity for cancer risk reduction [8, 9]. These reviews of the evidence, as well as multiple systematic reviews and meta‐analyses on this topic, have concluded that there is some evidence for a reduced risk of 11 different cancer sites when comparing the highest to the lowest levels of physical activity (Table 1).

**Table 1:** Summary of the observational epidemiologic evidence on the association between physical activity and cancer risk by cancer site. Summary evidence for the dose–response effect, biologic plausibility, and overall classification of evidence was acquired from the 2018 Physical Activity Guidelines Advisory Committee [8] and McTiernan *et al*. [10]. Risk reduction estimates were acquired from McTiernan *et al*. [10] and meta‐analyses previously conducted by the authors.

Cancer site	Overall classification of evidence	Approximate range of relative risk reduction for high versus low levels of physical activity	Evidence for dose–response effect	Biologic plausibility
Bladder	Strong	19–24%	Limited	Limited
Breast	Strong	19–27%	Yes	Yes
Colon	Strong	21–27%	Yes	Yes
Endometrial	Strong	19–29%	Yes	Yes
Esophageal adenocarcinoma	Strong	19–51%	Yes	Yes
Gastric cardia	Strong	15–19%	Yes	Yes
Renal	Moderate	12–16%	Limited	Yes
Lung	Moderate/Limited^a^	27–28%	Yes	Limited
Ovarian	Moderate	2–23%	Limited	Yes
Pancreas	Moderate	9–25%	Yes	Yes
Prostate	Limited	3–13%	Limited	Limited

^a^ Confounding by smoking is possible.

Specifically, there is strong evidence that physical activity reduces the risk of bladder, breast, colon, endometrial, esophageal adenocarcinoma, and gastric cancers. There is moderate evidence for an association between higher levels of physical activity with lower occurrence of renal, ovarian, pancreatic, and lung cancers. Nevertheless, confounding by tobacco smoking may exist for lung cancer. Limited evidence exists for an association between increased physical activity and decreased risk of prostate cancer. There is limited evidence for an increased risk of melanoma with higher physical activity levels. However, uncontrolled confounding by ultraviolet (UV) exposure in these studies may explain this possible increased risk. The evidence for an association between physical activity and other cancer sites remains insufficient.

The magnitude of the decreased risk associated with higher levels of physical activity ranges from about 10–25% for most of these cancer sites [10]. A dose–response association between increasing levels of physical activity and specific cancer risk is evident for several cancer sites but the methods for measuring and categorizing physical activity levels across epidemiologic studies have been inconsistent which precludes any definitive conclusions regarding the exact volume of physical activity that provide given levels of effect. Furthermore, insufficient evidence is available to determine if the association between physical activity and cancer risk varies by domain or type (i.e., aerobic versus resistance exercise) of physical activity [8].

Limited information exists at present on how the association between physical activity and cancer varies by cancer subtypes. With respect to how physical activity varies across population subgroups, there is evidence that being physically active is equally beneficial for men and women. Moreover, some evidence suggests that lifelong activity is particularly beneficial. However, activity later in life can also reduce cancer risk (e.g., activity done after menopause has been shown to reduce breast cancer risk irrespective of premenopausal activity) [8]. There is also evidence that physical activity benefits are comparable across all racial and ethnic groups.

## 3 Sedentary behavior and cancer incidence

Sedentary behaviors include all waking activities with an energy expenditure ≤ 1.5 metabolic equivalents of task (METs) performed in the sitting, reclining, or lying postures (e.g., watching television, working at a computer, sitting in a vehicle) [11]. It is important to recognize that high amounts of sedentary time are not synonymous with low levels of physical activity [12]. For example, an individual may achieve or exceed physical activity recommendations but also spend long, uninterrupted time sitting at a work computer and/or watching television at home. Conversely, a person may accumulate small amounts of sedentary time over a 24‐h period as a result of a physically demanding job, but also have no or low levels of recreational physical activity. Therefore, it is important to consider both the amount of time dedicated to physical activity and time spent in sedentary behavior for cancer prevention. The AICR developed an educational infographic to illustrate the importance of making time for physical activity and breaking up sedentary time for cancer prevention [13].

A recent update published by the 2018 PAGA Committee reported that there is moderate evidence to suggest that high levels of sedentary time are associated with an increased risk of colon, endometrial, and lung cancers, with limited evidence for a dose–response relation [14]. Recently published reviews also corroborate these findings [12, 15]. Specifically, high versus low levels of sedentary time were consistently associated with a range in relative risks (RR) of 1.28–1.44 for colon cancer, 1.28–1.36 for endometrial cancer, and 1.21–1.27 for lung cancer (Table 2).

**Table 2:** Summary of the observational epidemiologic evidence on the association between sedentary time and cancer risk by cancer site. Summary evidence was acquired from Jochem *et al*. [12].

Cancer site	Overall classification of evidence	Magnitude of relative risk increase for high versus low sedentary time	Evidence for dose–response effect	Biologic plausibility
Colon	Moderate	28–44%	Limited	Yes
Endometrial	Moderate	28–36%	Limited	Yes
Lung	Moderate^a^	21–27%	Limited	Limited

^a^ Confounding by smoking is possible.

Given that a high proportion of individuals spend the majority (~ 55%) of their time awake taking part in sedentary behaviors [16], interventions targeting reductions in sedentary time would contribute to reducing chronic disease risk, including cancer, at a population‐level. For individuals with habitually high levels of sedentary time, it is expected that replacing some of that time with light intensity or ambulatory activities (e.g., breaking up sedentary time by standing or walking) would lead to some health benefits, with the greatest benefits occurring when sedentary time is replaced with planned moderate‐vigorous intensity physical activity [14]. Additional research from prospective cohort studies is needed to assess the interactive effects of physical activity and sedentary time on cancer incidence [14]. Randomized controlled trials focused on promoting reductions in sedentary by replacing it with light, moderate, and/or vigorous intensity physical activity are also needed in individuals at high risk for cancer development.

## 4 Obesity and cancer incidence

Weight gain, which may eventually contribute to the development of obesity [body mass index (BMI) ≥ 30 kg·m^−2^], occurs when energy intake exceeds energy requirements from resting metabolic needs and physical activity energy expenditure. The prevalence of overweight (BMI ≥ 25 kg·m^−2^) and obesity (BMI ≥ 30 kg·m^−2^) in adults aged ≥ 18 years is 39% and 13%, respectively [17]. These stark overweight and obesity levels constitute a main determinant of the increasing prevalence of many cancer types that could surpass smoking as the main preventable cause of cancer [18]. Indeed, the International Agency for Research on Cancer (IARC) established that there is convincing evidence that excess body fatness (i.e., highest BMI category evaluated versus normal BMI of 18.5–24.9 kg·m^−2^) is associated with an increased risk of at least 13 different types of cancers (RRs ranging from 1.1 to 7.1), including endometrial, postmenopausal breast, colorectal, esophageal, renal/kidneys, meningioma, pancreatic, gastric cardia, liver, multiple myeloma, ovarian, gallbladder, and thyroid (Table 3) [19]. The WCRF/AICR also highlighted that there is convincing and sufficient evidence that obesity is associated with an increased risk of endometrial, esophageal, colorectal, liver, pancreatic, postmenopausal breast, and renal/kidney cancers [9]. Taken together, there is strong evidence that obesity is associated with cancers impacting digestive organs in men and women, as well as hormone‐sensitive organs/sites in women [20].

**Table 3:** Summary of the observational epidemiologic evidence on the association between obesity and cancer risk by cancer site. Summary evidence was acquired from Lauby‐Secretan *et al*. [19].

Cancer site	Overall classification of evidence	Magnitude of relative risk increase for BMI ≥ 25 versus BMI < 25	Evidence for dose–response effect	Biologic plausibility
Colorectal	Strong	10–30%	Yes	Yes
Gastric cardia	Strong	20–80%	Yes	Yes
Esophagus	Strong	15–480%	Yes	Yes
Liver	Strong	50–80%	Yes	Yes
Postmenopausal breast	Strong	10–12%	Yes	Yes
Gallbladder	Strong	20–60%	Yes	Yes
Endometrial	Strong	50–710%	Yes	Yes
Renal/kidney	Strong	30–80%	Yes	Yes
Meningioma	Strong/Moderate	40–213%	Limited	Limited
Pancreatic	Strong	20–50%	Yes	Yes
Multiple myeloma	Strong/Moderate	15–52%	Limited	Limited
Ovarian	Moderate	10–20%	Yes	Yes
Thyroid	Moderate	4–17%	Yes	Yes

Given the strong association between obesity/weight gain and cancer, it is assumed that weight loss may be a viable prevention approach for reducing cancer risk. A systematic review that included 34 studies reported that 16 of these studies found a statistically significant reduction in cancer risk in individuals who experienced weight loss [21]. Specifically, studies assessing the risk of cancer following bariatric surgery reported that patients who had received this surgery had a statistically significantly decreased risk of combined cancers when compared to controls with obesity (RRs ranging from 0.22 to 0.76) over a five to 12.5‐year follow‐up period [21]. Nonsurgical approaches to intentional weight loss defined as ≥ 9 kg weight loss since age 18 were also associated with a significant decrease in combined cancer incidence (RR = 0.88) over a 7‐year follow‐up period [21]. The observed benefits of weight loss on cancer risk were strongest in women (with mostly null findings in men) and most consistent for obesity‐related cancers [21]. Despite these findings, the impact of sustained long‐term weight loss and the increased risk of weight regain following weight loss, on cancer incidence, needs to be studied further to better inform weight loss strategies for cancer prevention. The avoidance of weight gain may also be a more viable target than sustained weight loss as a cancer preventive measure, given the several physiological alterations that persist beyond the initial weight loss period to promote weight regain [22]. These alterations include decreases in anorexigenic hormones such as leptin, increases in orexigenic hormones such as ghrelin, decreases in resting metabolic rate that are greater than what can be accounted for by changes in body weight (adaptive thermogenesis), increases in appetite sensations, and lower fat oxidation in the weight‐reduced state [22]. Further research is needed to provide evidence on various approaches to weight gain prevention and weight loss strategies for cancer prevention [20].

## 5 Biologic mechanisms linking physical inactivity, sedentary behavior, and obesity with cancer risk

**Fig. 1:**
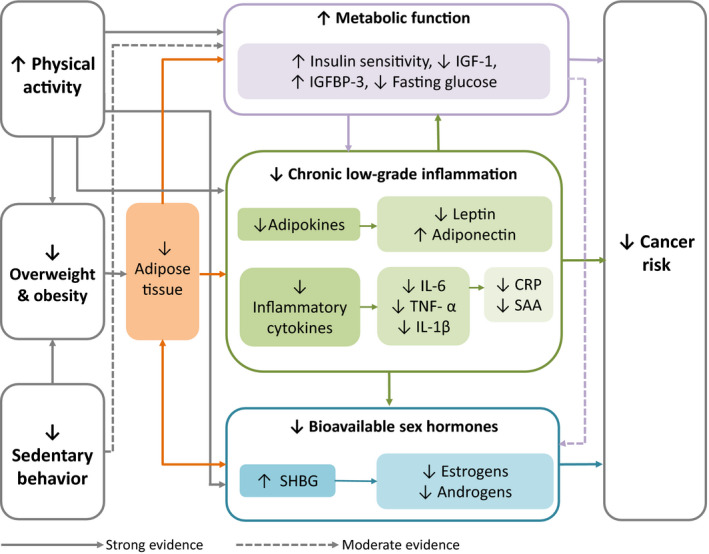
Hypothesized biologic mechanisms linking physical activity, excess body fat, and sedentary behavior to cancer risk. IGF‐1, insulin‐like growth factor‐1; IGFBP‐3, insulin growth factor‐binding protein‐3; IL‐6, interleukin‐6; TNF‐α, tumor necrosis factor‐α; IL‐1β, interleukin‐1β; CRP, C‐reactive protein; SAA, serum amyloid A; SHBG, sex hormone‐binding globulin.

Several hypothesized biologic mechanisms whereby obesity, physical activity, and sedentary behavior influence cancer risk are being elucidated through a combination of observational and experimental research studies (Fig. 1). The biologic pathways relating these exposures to tumorigenesis are incompletely defined and understood, but generally center on maintaining a healthy body weight, thereby reducing the risk of metabolic abnormalities, chronic low‐grade inflammation, and overstimulation of endogenous sex hormones. Evidence suggests that promoting physical activity and reducing sedentary behaviors can lead to cancer‐preventing health benefits through the above‐mentioned mechanisms, independently of body fat. Furthermore, the accumulation of ectopic fat tissue (i.e., the storage of triglycerides in areas outside of adipose tissue, such as the liver, skeletal muscle, the heart, and the pancreas) is of particular concern since it can interfere with normal cellular and organ function, thus increasing the risk for many chronic diseases including cancer [20, 23]. Finally, individual characteristics, such as age, sex, ethnicity/race, and genetics, as well as additional modifiable lifestyle factors (e.g., diet and smoking), may also modify the effects of physical activity, obesity, and sedentary behavior on these biomarkers and need to be considered when evaluating this evidence.

### 5.1 Metabolic function and insulin sensitivity

Insulin and insulin‐like growth factor (IGF)‐I are anabolic endocrine hormones with important physiological roles in glucose metabolism, as well as cell proliferation, cell death, and angiogenesis. Overstimulation of these biomarkers, their related binding proteins [i.e., insulin‐like growth factor‐binding protein (IGFBP)‐1 through ‐6], and their signaling pathways have been associated with increased risk of several malignancies such as breast, prostate, and colorectal cancers, but the exact molecular mechanisms by which this risk reduction occurs are not completely understood [20]. It is well established that excess body fat, particularly abdominal fat, is positively correlated with insulin resistance. When there are consistently high levels of blood glucose, excess insulin is secreted from the pancreas and commonly results in hyperinsulinemia leading to decreased IGFBP‐3 and subsequently increased levels of free IGF‐I, which may promote tumorigenesis [24]. In postmenopausal women, there is evidence that this pathway also modifies the bioavailability of sex hormones. Specifically, prolonged hyperinsulinemia reduces bioavailable sex hormone‐binding globulin (SHBG) and increases circulating estrogens and androgens, which may further contribute to tumorigenesis [24].

Modifiable lifestyle factors such as caloric restriction and physical activity are effective interventions for reducing adipose tissue and correcting metabolic abnormalities, thereby lowering the risk of certain cancers. Independently, and through its effect on adipose tissue, physical activity has been shown in observational epidemiologic studies and randomized controlled trials (RCTs) to reduce plasma insulin and increase insulin sensitivity and glucose metabolism [25]. Observational studies have also supported the hypothesis that physical activity lowers IGF‐1 levels and raises IGFBP‐3 levels [25]. However, findings from RCTs have failed to replicate these results, concluding that despite large reductions in weight and/or increased levels of physical activity, IGF‐1 bioavailability may not facilitate the relationship between obesity and cancer risk [26]. Finally, evidence is emerging that interventions targeting sedentary behavior, with or without physical activity, have small but statistically significant effects on insulin levels in adults [27].

### 5.2 Chronic low‐grade inflammation

Adipose tissue is a metabolic organ primarily composed of adipocytes that secrete an array of bioactive signaling molecules including pro‐inflammatory adipokines and cytokines that may stimulate cancer development. Leptin is an adipocyte‐derived hormone that informs the hypothalamus about the metabolic status of the body such that is suppresses appetite and increases energy expenditure when fat mass accumulates. However, consistently high levels of circulating leptin may contribute to ‘hyperleptinemia’ or leptin resistance in individuals with obesity, thus reducing the hypothalamus' response to leptin and current energy stores. In addition to reducing its impact on appetite and energy expenditure, this state of leptin resistance also perpetuates inflammation [28]. In contrast, adiponectin is an apoptosis‐inducing adipokine that is released by the adipocytes, but acts as an insulin‐sensitizing hormone by increasing glucose uptake and reducing triglyceride uptake in the muscle, and suppressing glucose production and triglyceride storage in the liver [28]. Adiponectin production is, however, reduced in individuals with obesity in response to increased production of pro‐inflammatory cytokines [e.g., tumor necrosis factor‐alpha (TNF‐α)], contributing to a state of ‘hypoadiponectinemia’ and increased tumorigenesis [28]. Finally, pro‐inflammatory cytokines themselves, including interleukin‐6 (IL‐6), IL‐1β, and TNF‐α, released by adipocytes increase the production of C‐reactive protein (CRP) and serum amyloid A (SAA) and may contribute to tumorigenesis [28, 29].

Generally, regular physical activity is thought to have anti‐inflammatory effects. Clinical studies have demonstrated that longer‐term physical activity interventions successfully reduce systemic levels of pro‐inflammatory biomarkers and increase levels of anti‐inflammatory biomarkers at least, in part, by decreasing adiposity [30]. There is a dearth of RCTs examining the inflammatory effects of sedentary behavior. Observational studies suggest that sitting time positively correlates with higher levels of adipokines and their related biomarkers, but these relationships are also likely mediated by adiposity levels [31].

### 5.3 Sex hormones

SHBG regulates the bioavailability of free estrogens, which if unbound, are considered to be highly active and associated with increased risk of some hormone‐sensitive cancers, particularly breast cancer. Observational studies and RCTs of women at risk of breast cancer have determined that higher levels of physical activity result in statistically significant reductions in estradiol, free estradiol, and estrone, while increasing levels of SHBG, regardless of menopausal status [30]. However, the evidence is stronger for postmenopausal than premenopausal women [30]. Furthermore, there is growing support for the hypothesis that body fat loss is mainly responsible for mediating the effect of physical activity on sex hormones [30, 32]. Evidence from studies of cancer‐free women suggests that interventions combining caloric restriction with physical activity are most effective to produce favorable changes in endogenous sex hormones [33].

Androgens, produced primarily in men and to a lesser extent in women, have also been implicated in tumorigenesis. In the male prostate, androgen and androgen receptors regulate the rate of cell growth and death, and are closely involved with the development of prostate cancer [34]. However, epidemiologic studies of the relationship between androgen levels and prostate cancer risk have been inconsistent [35]. There is also evidence that the androgen‐signaling pathway influences breast carcinogenesis, but the direction of effect differs among clinical and observational research [36]. Physical activity and obesity have both been investigated as factors that may affect androgen levels in men and women. In men, there appears to be a strong negative correlation between adiposity and free, bioavailable, and total testosterone, but the relationship with physical activity remains inconclusive [25]. In women, obesity corresponds with excess levels of androgens and physical activity is associated with small, but statistically significant reductions in free testosterone and other androgens [32, 37].

### 5.4 Emerging hypotheses

There are a number of additional biologic mechanisms related to physical activity, obesity and sedentary behavior under active investigation for their role in cancer development. The biologic relevance of these pathways for cancer has been supported by experimental findings, yet they lack, or are inconsistent with, the epidemiologic evidence required to support them more convincingly. Discrepancies may be the result of random error or systematic biases arising from the use of self‐report measures of physical activity or sedentary behavior, and the challenges inherent in laboratory analyses of some of these other pathways [15].

Physical activity is hypothesized to affect the balance between reactive oxygen species (ROS) and antioxidant defenses that can result in oxidative stress [30]. ROS may cause chromosomal abnormalities, DNA damage, and mutations in tumor‐suppressing genes. Acute exercise appears to promote oxidative stress and a pro‐oxidant environment but as physical activity is repeated, adaptations to this stress occur and eventually antioxidant defenses are built up [30, 38]. Correspondingly, individuals with obesity exhibit lower levels of antioxidants and higher levels of oxidative stress, which may also decrease insulin sensitivity and lead to insulin resistance [39].

A similar pattern emerges from the relationship between physical activity and immune function, whereby the body responds differently to acute and prolonged bouts of exertion [40]. Bouts of unusually heavy and/or long exertions (e.g., running a marathon) can lead to transient immune dysfunction, while shorter duration aerobic physical activity stimulates short‐term increases in immunoglobulins, neutrophils, natural killer cells, cytotoxic T cells, and immature B cells, which over time, enhance immunosurveillance [40]. These findings are particularly relevant to individuals with impaired immunity, including older adults and individuals with obesity [25].

Physical activity may affect the development of cancer through epigenetic alterations to chromosomes, DNA methylation, expression of microRNA, and chromatic structure [25]. Telomere length, a prognostic marker of aging and disease, has been shown to be longer in men with healthy eating and exercise habits [25]. Part of this association may be explained by damage caused to telomere length by ROS [41]. Altered patterns of DNA methylation, considered to be a hallmark of cancer for its regulation of tumor suppressor genes and oncogenes, continues to be studied as a possible link between obesity and cancer risk [42, 43]. In observational studies, higher self‐reported physical activity was associated with a favorable increase in a surrogate marker of global DNA methylation [41]. Thus far, the association between global DNA methylation and obesity is inconsistent.

Emerging evidence suggests that an altered intestinal microbiome may explain some of the association between obesity and cancer, as microbiota may produce cancer‐promoting metabolites, or promote inflammation and insulin resistance [20]. Obesity‐related inflammation originates in the intestinal lumen, where bacteria‐derived substances leak into the bloodstream and are thought to initiate inflammation [20]. Toxic metabolites produced in response to obesity and a high fat diet appear to cause DNA damage through the formation of ROS. In support of this hypothesized pathway, recent systematic reviews of observational studies in humans have demonstrated that individuals with obesity have a different microbial profile than lean individuals and that microbial dysbiosis (or ‘imbalance’) is associated with colorectal cancer [44, 45].

## 6 Recommendations for future epidemiologic research directions

Significant knowledge gaps remain in our understanding of the biologic pathways that link physical inactivity, sedentary behavior, and obesity with increased cancer risk. These gaps highlight potential directions of future research (Table 4). Additional systematic reviews and meta‐analyses are needed to pool and strengthen the evidence for cancer sites for which the evidence is currently limited or emerging (Tables 1, 2, 3). In addition to emerging evidence on the link between physical activity, sedentary behavior, and obesity with several types of cancer, accumulating evidence shows that race/ethnicity, age, and socioeconomic status, among other demographic characteristics, can have profound impacts on these risk factors for cancer incidence and the biologic pathways involved [46]. Despite this evidence, the majority of studies do not assess effect modification by these key demographic characteristics, while including a large number of participants who have high socioeconomic status, education, and/or are White. Therefore, findings may lack generalizability to more diverse and minority populations [46] who may gain the most benefit from lifestyle modifications on reducing cancer risk. Addressing barriers to participation in clinical trials for minority populations (i.e., mistrust, experimentation fears, low socioeconomic status, logistical barriers) could serve to improve health outcomes and reduce public and private medical expenditures in these populations [47].

**Table 4:** An outline of recommendations for future research directions.

Exposure type (physical activity, sedentary behavior, obesity, or all)	Recommendations for future research directions/studies
All	Study effect modification by age, race/ethnicity, socioeconomic status
All	Conduct pooled analyses, meta‐analyses and large prospective cohort studies for cancer sites with limited or unassignable grades of evidence
Physical activity and sedentary time	Include both self‐report and device‐based measures of physical activity and sedentary time to improve quantification of these behaviors
Physical activity and sedentary time	Examine the dose–response associations between physical activity and sedentary time with cancer risk
Physical activity	Assess the association between different parameters of activity (frequency, intensity, type, duration, and volume) on cancer incidence
Physical activity	Assess the effects of different exercise prescriptions varying in intensity, type, duration, volume, and progression on biomarkers for cancer incidence
Sedentary time	Examine the association between sedentary time and cancer risk for cancer sites for which the evidence is currently limited or unavailable
Sedentary time	Assess effects of reducing sedentary time on biologic markers of cancer risk
Sedentary time	Assess role of standing and breaking up sedentary time on cancer risk
Obesity	Use direct quantification of excess body fat and body fat distribution (e.g., waist and hip circumferences, visceral and subcutaneous abdominal fat mass, total fat mass)
Obesity	Assess body weight change and weight loss in behavioral interventions (diet and/or physical activity interventions)
Obesity	Assess how weight gain and/or weight loss influence biomarkers for cancer risk

Moreover, future studies should use both self‐report and device‐based (e.g., accelerometry) measurement tools to improve the quantification of physical activity and sedentary behaviors, as well as provide context to these behaviors. Additionally, studies are needed to provide evidence on the associations between characteristics of physical activity (frequency, intensity, type, duration, and volume) and sedentary time (standing time and breaks in sedentary behaviors) with cancer incidence and intermediate biomarkers for cancer risk. These results could then be used to inform the design and conduct of RCTs targeting changes in one or more of these physical activity and/or sedentary behavior characteristics. For example, the Breast Cancer and Exercise Trial in Alberta (BETA) aimed to assess the effects of different exercise durations on biomarkers for postmenopausal breast cancer risk in 400 postmenopausal, previously inactive women. These women were randomized to either 150 or 300 min per week of aerobic exercise for 1 year [48]. The study concluded that higher doses of physical activity are superior to the recommended dose for reducing adiposity, but not for improving other biomarkers of insulin resistance, inflammation, or endogenous sex hormones [48, 49]. Additional RCTs, similar to BETA, that focus on comparing other components of an exercise prescription, as well interventions that focus on reducing sedentary time or on biomarkers for cancer risk would add to this literature.

Lastly, more studies are needed to improve understanding of the etiologic role of excess body fat, rather than body weight, on cancer risk by including measures other than BMI, such as waist and hip circumferences, visceral and subcutaneous fat mass, and total fat mass. Furthermore, prospective cohort studies that focus on the role of body weight change and weight loss in cancer prevention, in addition to RCTs that assess the impact of behavioral interventions (diet and/or physical activity interventions) targeting weight loss on biomarkers for cancer risk, are needed. Observational studies and RCTs could also assess the impact of the rate of weight gain and/or weight loss on biomarkers for cancer incidence, as these results could be used to inform the ‘intensity’ of behavioral prescriptions needed to induce weight loss or prevent weight gain for cancer prevention.

## 7 Conclusion

In this review, we summarized the epidemiologic evidence relating physical activity, obesity, and sedentary behavior with cancer incidence and described established and emerging pathways that support the biologic plausibility of these relationships. Currently, there is strong evidence that physical inactivity and obesity independently increase the risk of multiple cancers, and some evidence that sedentary behavior has a similar effect. Additional research is needed to increase the depth and scope of knowledge pertaining to these associations.

Globally, high rates of physical inactivity, excess body fat, and sedentary time contribute substantially to the development of noncommunicable diseases including cancer. High BMI, in particular, is a risk factor that continues to increase in prevalence, even in developing countries [50]. Changes to the food environment, including the marketing and availability of energy‐dense foods, and increasing wealth may be primary drivers of this trend [50]. Increases in urbanization, sedentary jobs, and leisure‐time spent at the computer or watching television have further led to inactive lifestyles and increase the risk of multiple noncommunicable diseases [50]. Through translation and dissemination of research, public health organizations and primary healthcare providers can increase awareness and promote healthy behaviors that reduce the overall burden of cancer.

## Conflict of interest

The authors declare no conflict of interest.

## Author contributions

CMF, CR‐B, and JM contributed equally to the conceptualization and writing of this review.
